# Neutral Poly-/perfluoroalkyl Substances in Air and Snow from the Arctic

**DOI:** 10.1038/srep08912

**Published:** 2015-03-09

**Authors:** Zhiyong Xie, Zhen Wang, Wenying Mi, Axel Möller, Hendrik Wolschke, Ralf Ebinghaus

**Affiliations:** 1 Helmholtz-Zentrum Geesthacht, Centre for Materials and Coastal Research, Institute of Coastal Research, Department for Environmental Chemistry, Geesthacht, 21502, Germany; 2 National Marine Environmental Monitoring Center, Dalian 116023, China

## Abstract

Levels of neutral poly-/perfluoroalkyl substances (nPFASs) in air and snow collected from Ny-Ålesund were measured and their air-snow exchange was determined to investigate whether they could re-volatilize into the atmosphere driven by means of air-snow exchange. The total concentration of 12 neutral PFASs ranged from 6.7 to 39 pg m^−3^ in air and from 330 to 690 pg L^−1^ in snow. A significant log-linear relationship was observed between the gas/particle partition coefficient and vapor pressure of the neutral PFASs. For fluorotelomer alcohol (FTOHs) and fluorotelomer acrylates (FTAs), the air-snow exchange fluxes were positive, indicating net evaporative from snow into air, while net deposition into snow was observed for perfluorooctane sulfonamidoethanols (Me/EtFOSEs) in winter and spring of 2012. The air-snow exchange was snow-phase controlled for FTOHs and FTAs, and controlled by the air-phase for FOSEs. Air-snow exchange may significantly interfere with atmospheric concentrations of neutral PFASs in the Arctic.

Poly-/perfluoroalkyl substances (PFASs), including neutral PFASs and perfluoroalkyl acids (PFAAs), have been widely produced in increasing quantities for several decades, and are raising concern because of their environmental persistence, bioaccumulative potential and their possible adverse effects on humans and wildlife^1^,^2^,^3^,^4^. The contamination of various environmental media with PFAAs has been observed even in remote regions, such as the Arctic^5^,^6^. Due to the low volatility and high water solubility of PFAAs in their anionic form, they are not prone to long range atmospheric transport (LRAT)^7^,^8^,^9^, which is considered as the primary transport way for traditional persistent organic pollutants (POPs) into remote regions^10^. A hypothesis was proposed that precursors, such as neutral PFASs, reach remote sites via LRAT from their point(s) of emission and are subsequently oxidized to PFAAs in the atmosphere^11^.

Previous studies showed that PFAAs in the environment could be derived from the degradation of neutral PFASs, such as fluorotelomer alcohols (FTOHs) and fluorotelomer acrylates (FTAs)^12^,^13^, and the source of perfluorooctane sulfonate (PFOS) could be atmospheric oxidation of volatile perfluorooctane sulfamido alcohols, such as perfluorooctane sulfonamides (FOSAs) and perfluorooctane sulfonamidoethanols (FOSEs)^11^,^14^. Piekarz et al. indicated that the atmospheric residence time of FTOHs was about 50 days due to their low reaction rates with hydroxyl radicals^15^. This is enough time for possible regional and global atmospheric transport, even to the Arctic^16^,^17^. Thus, the gas/particle partitioning of such volatile precursors may become an important factor influencing their LRAT potential and global distribution.

Snow is an important part of cold environments, especially in the Arctic. The high efficiency of snow as a scavenger of both vapor and particle POPs from the atmosphere has received growing attention due to the large surface area of snow and enhanced surface sorption under subzero temperatures. Due to trace levels of POPs in the Arctic atmosphere, a minor change of their input or output to the atmosphere may result in considerable perturbations^18^. Daly and Wania simulated the influence of snow on the fate of several typical POPs and found a notable effect on the predicted seasonal air concentrations, especially for volatile compounds^18^. Herbert et al. observed rapid changes in PCBs and OC pesticide concentrations in Arctic snow, and the complicated re-emission process from snow to the overlying atmosphere^19^. Thus, studies investigating the air-snow exchange and the flux of POPs are essential for understanding their multi-media distribution and their fate in cold environments, such as the Arctic. However, to date, such studies on air-snow exchanges and fluxes of PFASs are limited.

The primary objectives of the present study were to investigate the occurrence of neutral PFAS compounds (Table S1) in the atmosphere and snow in the Arctic, and to estimate the air-snow exchanges and fluxes of neutral PFASs and the key influencing factors. To our knowledge, this is the first study on the concentration of neutral PFASs in Arctic snow and their air-snow exchange.

## Results

### Neutral PFASs in Arctic air

**Figure 1:**
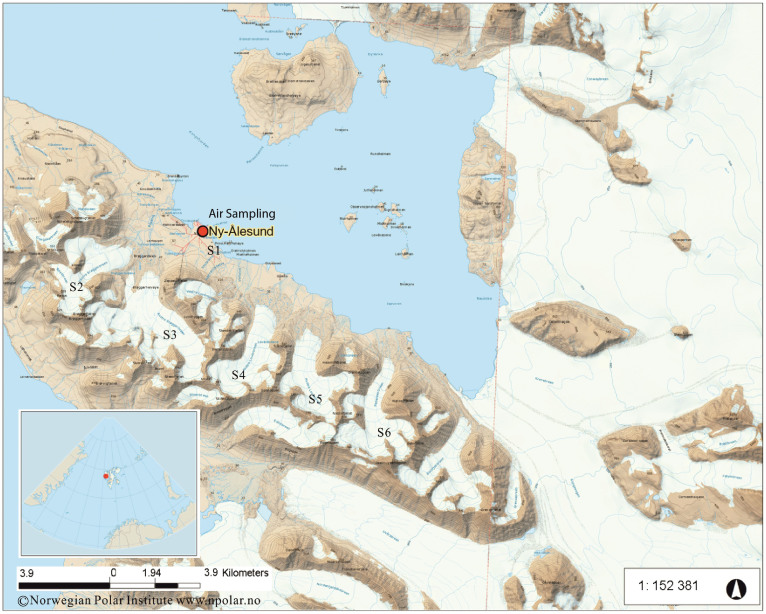
Sampling Station Map. Map for air sampling at Ny-Ålesund (78°55′N, 11°56′E, Sep. 2011–Sep. 2012), and snow sampling stations (Jan. and May 2012) in the Arctic. The glaciers for snow sampling S1: Ny-Ålesund; S2: Vestre Brøggerbreen; S3: Austre Brøggerbreen; S4: Lovèn breane; S5: Austre Lovènbreen; S6: Pedersenbreen (The Map is created using the Norwegian Polar Institute's free map product licensed under the Creative Commons Attribution 4.0 International (CC BY 4.0) license).

**Figure 2:**
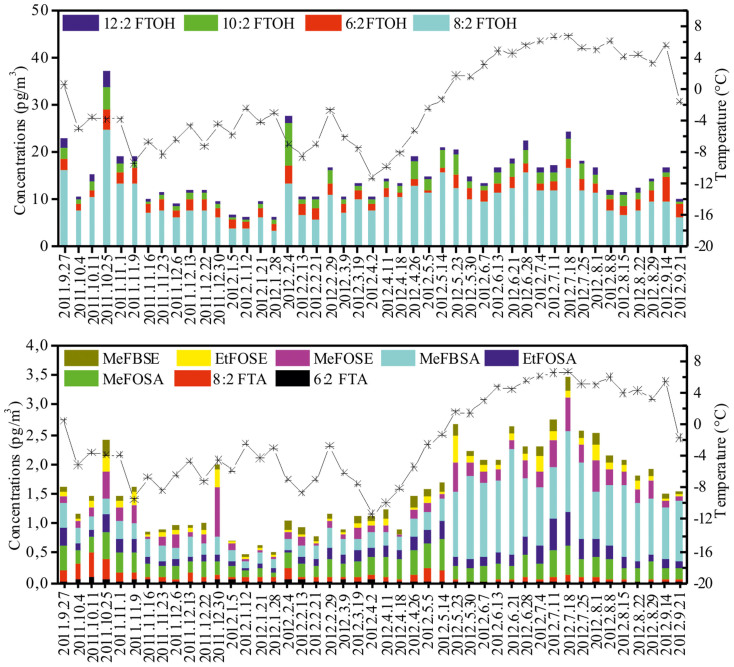
Temporal distribution of neutral PFASs. FTOHs and other neutral PFAS compounds measured in air at Ny-Ålesund, in the Arctic from Sep. 2011 to Sep. 2012. The black line indicates average air temperatures.

**Figure 3:**
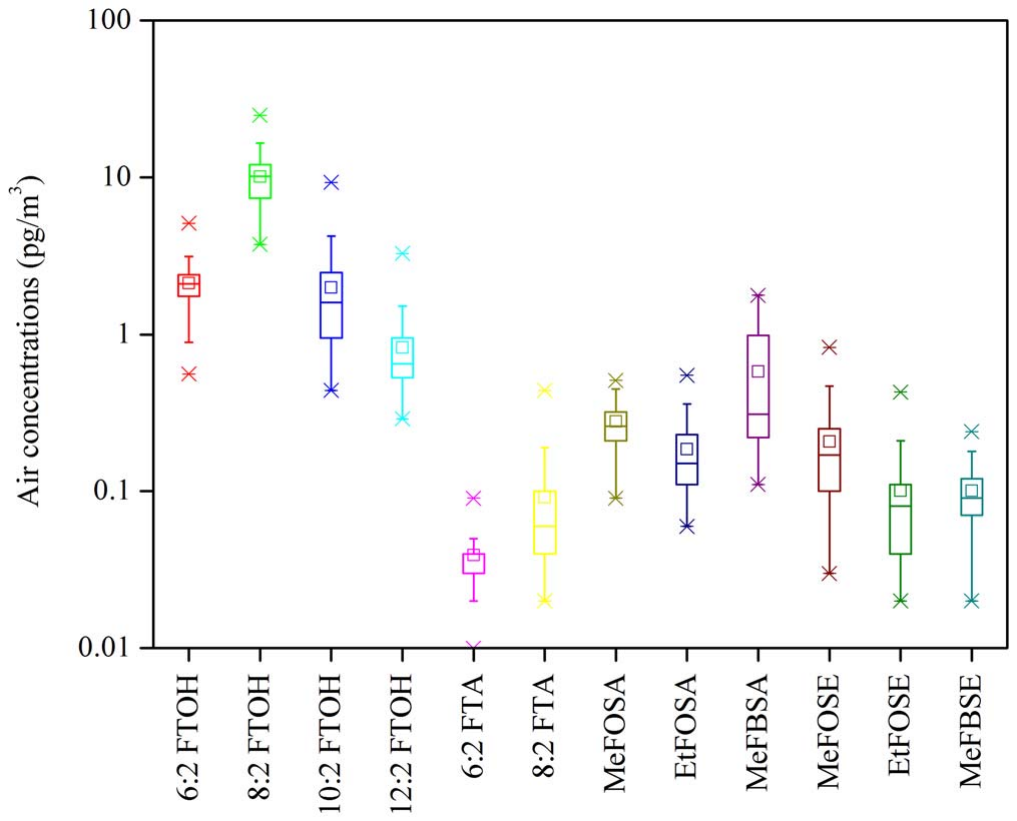
Atmospheric concentration of neutral PFASs. Graph indicating the concentration of the neutral PFASs in air collected from Ny-Ålesund, the Arctic.

High-volume air sampling (gas and particle phases) was carried out from September 2011 to September 2012 on a platform for atmospheric observation at the German station located in Ny-Ålesund (78°55′N, 11°56′E) (Figure 1, Table S2). A summary of the data for neutral PFASs concentrations in the Arctic atmosphere is given in Figure 2 and Table S3. The total concentration (vapor plus particle phases) of the 12 neutral PFASs (ΣnPFASs) in the Arctic atmosphere ranged from 6.7 to 39 pg m^−3^ (mean: 17 pg m^−3^). Figure 3 shows the concentration range, mean and media of individual neutral PFASs in Ny-Ålesund. Gaseous PFASs were dominant in all air samples and accounted for 91% of ΣnPFASs. The fraction of each neutral PFAS compound to ΣnPFASs is believed to be related the specific vapor pressure of each PFAS and will be discussed later. The concentrations of PFASs were comparable with the data reported by Shoeib et al., who investigated the atmospheric levels of neutral PFASs in the North Atlantic and Canadian Archipelago^20^. As expected, the measured concentrations were considerably lower than those of urban and semi-urban areas^21^,^22^. The most abundant chemical in air is 8:2 FTOH, representing 61% of ΣnPFASs, and next three most abundant compounds were 6:2 (13%), 10:2 (12%) and 12:2 FTOH (4.8%) (Table S3). The concentrations of 6:2, 8:2, 10:2 and 12:2 FTOH (ΣFTOHs) in air varied from 5.6 to 34 pg m^−3^ with a mean value of 14 pg m^−3^ (Figure 3), which is lower than those in Toronto (mean 79.5 pg m^−3^)^21^, Hamburg (from 32 to 204 pg m^−3^)^22^, and the North Sea (84 pg m^−3^)^23^. The concentrations of MeFBSA, MeFOSA and EtFOSA (ΣFOSAs) in air were higher than the levels of MeFBSE, MeFOSE and EtFOSE (ΣFOSEs), which is in agreement with the results reported by Wang et al.^24^.

The average ratio of 8:2 to 10:2 to 6:2 to 12:2 FTOH in air obtained in this study was 4.8:0.9:1.0:0.4. The ratio can be considered as an indicator of long range atmospheric transport due to the longer atmospheric residence time of FTOH 8:2 (80 d) than that of FTOH 6:2 (50 d)^15^. Higher ratios were measured in the Arctic air compared with urban/-semi-urban areas, such as 1.1:0.2:1.0 for 8:2 to 10:2 to 6:2 FTOH in semi-urban location in Toronto, Canada^21^, and 1.8:0.6:1.0 in the southeast of Hamburg, Germany^22^, while the ratios were similar to the reported at remote sites, such as in the Mount Bachelor Observatory^15^, the Canadian Arctic^6^ and the North Sea^23^. The results confirmed that the FTOHs in Arctic air were from long range atmospheric transport process.

To investigate the relationship between the variation of gas-particle partitioning of PFASs in the atmosphere and vapor pressure, the linear relationships between log*K*_SP_ and log*p*°_L_ for all the 45 air samples were estimated. The physicochemical calculator SPARC v4.6 (October 2011 release w4.6.1691–s4.6.1687) was employed to calculate the values of *p*°_L_ for individual PFASs at different sampling temperatures (Table S2). Considering the p*K*_a_ values of 6:2 and 8:2, FTA were below the environmentally relevant pH range (≤7) and they mainly exist as anions in aerosols as stated by Wang et al.^24^. The corrected log*K*_SP_ (neutral form) of 6:2 and 8:2 FTA were considerably lower than those of FTOHs, FOSEs and FOSAs with similar vapor pressures, thus, they were excluded in the regression calculation performed in this study. The comprehensive regression results show that log*K*_SP_ correlates well with log*p*°_L_ (Figure S1). Table S2 presents the slopes, intercepts, relationship coefficients (*r*^2^) and significance levels (*p*) of all the 45 samples. The slopes and intercepts ranged from −0.63 to −0.15 (mean: −0.37) and from −2.57 to −1.31 (mean: −1.90), respectively. The results confirm that the gas-particle partitioning of neutral PFASs (e.g., FTOHs, FOSAs and FOSEs) followed the classical log*K*_SP_ - log*p*°_L_ relation for classic POPs^24^.

### Neutral PFASs in Arctic snow

**Figure 4:**
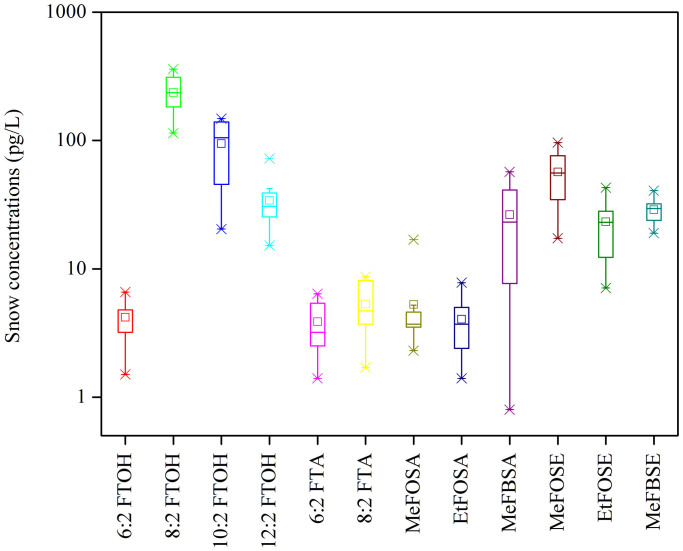
Neutral PFASs concentrations in Arctic snow. Graph indicating the concentration of the neutral PFASs in snow samples collected from Ny-Ålesund, the Arctic.

Snow sampling was conducted on glaciers around Ny-Ålesund during January and May 2012 (Figure 1, Table S4). The concentrations of neutral PFASs in the Arctic snow samples are summarized in Table S3 and Figure 4. The range of the total concentration was from 334 to 692 pg L^−1^ with an average value of 523 pg L^−1^. To our knowledge, there are few reports on PFAS concentrations in snow, thus it is hard to compare these values with those from other regions. Similar to the situation in air, 8:2 FTOH is the dominant species in the Arctic snow, accounting for 45% of the total concentration. ΣFTOHs in snow were between 218 and 507 pg L^−1^ (mean: 369 pg L^−1^). The composition of the 12 PFASs in snow was different to that in air. Besides 8:2 FTOH; 10:2 FTOH, MeFOSE and 12:2 FTOH were the three most abundant species, representing 18%, 11% and 6.5% of the ΣPFASs in snow, respectively (Table S3). The difference between the compositions in air and snow might be caused by different degradation processes for neutral PFASs in air and snow, and different air-snow exchange potentials of individual chemicals^23^. The average ratio of 8:2 to 10:2 to 6:2 to 12:2 FTOH was 56.2:22.5:1.0:8.1 in snow. However, to date, there has been few data such ratios in snow, thus it is hard to compare the variation between Arctic and urban areas. The ratios in snow are considerably higher than those in air, and could be explained by the higher volatilization potential of 6:2 FTOH and by different degradation processes of FTOHs in air and snow.

### Air-snow exchange fluxes of PFASs in the Arctic

**Figure 5:**
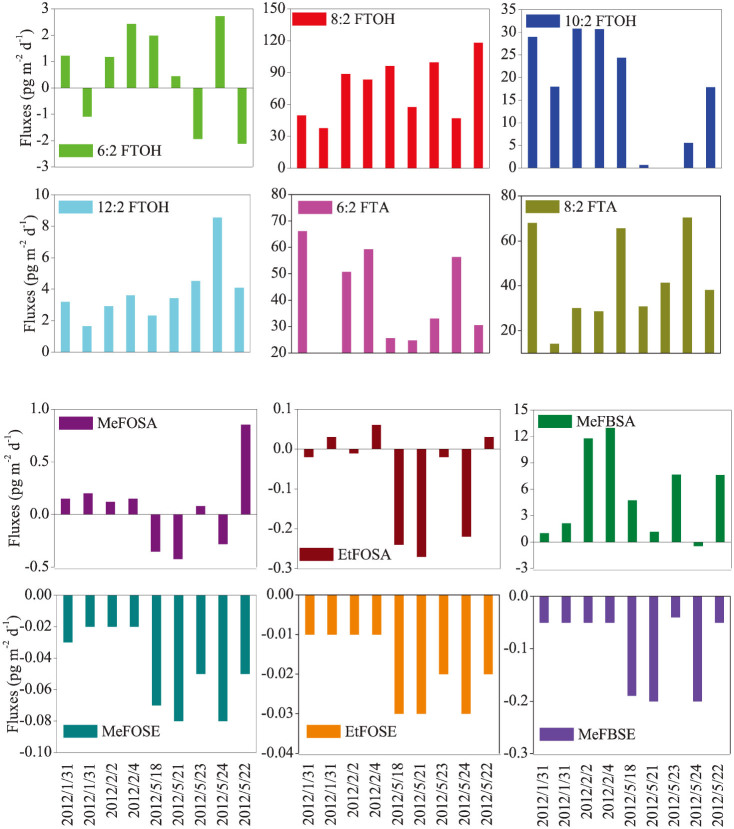
Air-snow exchange fluxes. Estimated air-snow exchange fluxes (pg m^−2^ d^−1^) for the 12 neutral PFASs. Negative fluxes represent net deposition into snow and positive values are net volatilization fluxes into the atmosphere.

Figure 5 shows the calculated net fluxes between air and snow for the 12 neutral PFASs from September 2011 to September 2012, whereby negative fluxes represent net deposition into snow and positive values are net volatilization fluxes into the atmosphere. It should be noted that the dominant trends of air-snow exchange were different among the various neutral PFASs species (Table S5). For FTOHs and FTAs, the total air-snow exchange fluxes were positive, and the fluxes were negative for FOSEs. The highest positive and negative fluxes were observed for 8:2 FTOH (with a value of 118 pg m^−2^ d^−1^) and 6:2 FTOH (−2.1 pg m^−2^ d^−1^), respectively. However, 6:2 FTOH and FOSAs illustrated changing directions of air-snow exchange driven by varying concentration gradients between air and snow. Uncertainty of the parameters involved in the calculation of exchange fluxes would influence the flux values. To investigate the influence of the uncertainties in *C*_s_, *C*_a_ and SSA on the exchange fluxes and direction, the uncertainties of *C*_s_, *C*_a_ and SSA were assumed to be 100%, and the exchange fluxes were calculated based on the Monte Carlo method using Software Oracle Crystal Ball 11.1. The cumulative frequency of Monte Carlo analysis (10 000 times) for the 12 PFAS compounds are presented in Figure S2. The results indicate that the exchange direction did not change with 95% certainty for all neutral PFASs except for 6:2 FTOH and EtFOSA.

The evident variability in air-snow exchange fluxes can be attributed to the varying *K*_SA_ values of the different neutral PFAS species, which results in their potential of retaining in snow. *K*_SA_ is a useful parameter expressing how much of the PFASs can be retained in snow under equilibrium conditions with air, and is determined by a combination of snow and compound specific properties. As stated by Hansen et al., snowmelt and the increased temperature would lead to a decrease of *K*_SA_^25^. Considering all collected snow samples in this study were fresh, the physical properties of snow can be considered as being constant, thus only the compound properties would influence the values of *K*_SA_. Daly and Wania found that volatilization from snow is not a significant for chemicals with log*K*_OA_ values higher than 10 (or log*K*_SA_ > 0.5) at 25°C^18^. Accordingly, PFASs with a log*K*_SA_ > 0.5 would is unlikely to be volatile in snow, and as expected, FOSEs (log*K*_SA_ > 0.5) have a negative exchange fluxes between air and snow (Table S6).

## Discussion

Previous studies have confirmed that POPs in snow were mainly from the atmosphere, and that snow has a high efficiency in scavenging vapor and particle-bound POPs out of the atmosphere due to their large surface area and the low temperatures promoting surface sorption. The positive FTOHs and FTAs fluxes observed in this study show the dynamic nature of their air-snow exchange, i.e., re-volatilization from the snow occurs rapidly following their wet deposition, which implies their strong volatilization potential. Compared with FTOHs, FTAs and FOSEs, the net air-snow exchange fluxes for FOSAs varied between deposition and volatilization (Table S5), which implies that FOSAs in air and snow are in dynamic equilibrium.

In theory, under conditions of increasing temperature and snowmelt, snow would lose capacity to hold sparingly water-soluble chemicals due to decreases in volume, *A*_snow_, and values of log*K*_SA_^25^. As a result, chemicals accumulated in snow would have the potential for volatilization. Daly and Wania indicated that the air concentration of semi-volatile organic pollutants could be considerably influenced by snowmelt, and a high concentration would be observed during or after snowmelt, especially in high latitude areas^18^. A significant correlation in this study was observed between the ambient temperature and the atmospheric concentrations of FOSEs and FOSAs (*r*^2^ = 0.70, *p* < 0.01) (Figure S3). However, the concentrations of FTOHs and FTAs did not show a significant correlation with the ambient temperature. The differences in behavior of the different chemicals are believed to be due to differences in their volatilization potential in snow as discussed above. Once FTOHs and FTAs have being scavenged into snow, they have strong volatilization potential to air. On the other hand, net volatilization of FOSEs and FOSAs out of snow occurs only under conditions of increasing temperature or snowmelt (and hence lowers *K*_SA_). As a result, air concentrations of FOSEs and FOSAs are significantly influenced by temperature. Hansen et al. found that the increase in atmospheric hexachlorocyclohexane isomers concentrations were more pronounced than those for fluorene, phenanthrene and PCB-28 toward the end of the winter in the Canadian High Arctic^25^. In general, chemical fluxes between snow and air are highly dynamic. Those chemicals having higher log*K*_SA_ values can be efficiently retained in snow, which could lead to an increase in their concentration in the atmosphere during or after snowmelt.

## Methods

### Air and snow sampling

Air sampling was conducted on the German atmospheric observation platform located in Ny-Ålesund (78°55′N, 11°56′E) (Figure 1), Svalbard from September 27, 2011 to September 21, 2012, and a total of 45 air samples were collected. A glass fiber filter (GFF: diameter, 150 mm, pore size, 0.7 μm) and a self-packed polyurethane foam (PUF)/XAD-2 cartridge (PUF: *φ*5.0 cm × 2.5 cm; 35 g XAD-2, particle size: 0.3–1.0 mm) were simultaneously employed for collecting air samples (gas- and particle-phase) using a high-volume air sampler (Figure S4). Each sample was collected at ~15 m^3^ h^−1^ for 7 days to obtain a sample volume of ~2500 m^3^. After sampling, cartridges and GFFs were separately sealed in air tight aluminum bags and stored at 4°C and −20°C prior to extraction, respectively. Field blanks consisted of GFFs and PUF/XAD-2 cartridges that were taken to the field site and handled in the same manner as real samples. Detailed information on the sampling dates, air volume, total suspended particulate and the average temperature for each sample are listed in Table S2.

Snow sampling was conducted around Ny-Ålesund from January to May 2012 (Table S4). The sampling depth was 0–5 cm and the samples were collected within 4 h after snowfall, thus the snow samples can be considered as fresh. Snow was collected using a pre-cleaned 40 L stainless steel barrel. After collection, the snow was allowed to melt within 24 h in the lab (at ~15°C). Around 5–8 L melt water was extracted with a polymer resin cartridge (40 g PAD-3 packed in a glass column) at a flow rate of ~300 mL min^−1^, and was used for the determination of neutral PFAS. PAD-3 columns were stored at 4°C and the GFF filters were stored at −20°C.

### Sample extraction

Prior to extraction, internal standards mixture (IS, 2.5 ng) (^13^C 6:2, 8:2 and 10:2 FTOH, MeFOSA D3, EtFOSA D5, MeFOSE D7 and EtFOSE D9) was spiked into a PUF/XAD-2 cartridge and GFFs, respectively. A modified Soxhlet apparatus (MX extractor) (Figure S5), specially designed for the self-packed PUF/XAD-2 and PAD-3 column, was employed to extract PUF/XAD-2 and GFF for 16 h using dichloromethane (DCM). Extracts were evaporated to 5 mL with hexane as a keeper and 3 g Na_2_SO_4_ was added to remove residual water. Samples were further reduced to 200 μL under a gentle stream of nitrogen and spiked with 1 ng of 9:1 FTOH as an injection standard.

### Instrumental analysis

The 12 neutral PFASs were analyzed using an Agilent 6890 gas chromatograph - 5973 mass spectrometry that was equipped with a 60 m SUPELCO WAX® 10 column (60 m × 0.25 mm × 1.0 μm). Measurements were made using a selective ion monitoring mode with positive chemical ionization (PCI). Methane was used as a reagent gas for PCI and helium (at a flow rate of 1.3 mL min^−1^) was used as carrier gas. The detailed instrumental parameters are presented in SI. The full names and other information of the 12 neutral PFASs determined in the present study are summarized in Table S1.

### Quality Control

Detailed quality control processes and breakthrough tests for air samples were described elsewhere^23^,^24^, Method detection limits (MDLs) for neutral PFASs in air and snow samples were derived from mean blank values plus three times the standard deviation (Table S1). All results were recovery corrected for neutral PFASs with corresponding internal standards.

### Calculation method of air-snow exchange flux

The air-snow exchange fluxes for the PFASs were calculated based on the modified Whitman two-film resistance model, the processes and methods are described in detail in the supplementary information. Briefly, the air-snow exchange flux (*F*_gas_, pg (s m^2^)^−1^) was calculated as^25^:
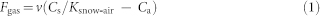
where *C*_s_ (pg m^−3^) and *C*_a_ (pg m^−3^) are the concentrations of target compounds in snow and air, respectively, *v* is an exchange velocity (m s^−1^), *K*_snow-air_ is the snow-air partition coefficient and can be expressed by the following equation^25^:
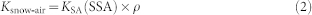
where *K*_SA_ is the snow interface-air partition coefficient, SSA is the specific surface area (m^2^ kg^−1^), and *ρ* (kg m^−3^) is the density of water.

As stated above, all snow samples collected in this study were fresh, thus, the snowpack can be considered to be homogeneous. Eqs. (1) and (2) show that snow physical parameters are key factors influencing *F*_gas_. In this study, snow physical parameters were used and/or calculated based on the empirical relationship developed by Legagneux et al.^26^. The overall or summation solute hydrogen bond acidity (Σ*α*_2_^H^) and the overall or summation solute hydrogen bond basicity (Σ*β*_2_^H^) were calculated according to Lyakurwa et al.^27^, and the solute gas-hexadecane partition coefficient (log*L*^16^) was calculated using SPARC software.

## Author Contributions

Z.X. and R.E. conceived the study; Z.X., A.M. and H.W. carried out the sampling; W.M. and Z.X. performed sample analysis; Z.W. and Z.X. conducted data management and wrote the manuscript with significant contributions from all co-authors.

## Supplementary Material

Supplementary Information Supplementary Information

## References

[b1] Houde M., De Silva A. O., Muir D. C. & Letcher R. J. Monitoring of perfluorinated compounds in aquatic biota: an updated review. Environ Sci Technol 45, 7962–73 (2011). 21542574 10.1021/es104326w

[b2] Kannan K. Perfluoroalkyl and polyfluoroalkyl substances: current and future perspectives. Environ Chem 8, 333–8 (2011).

[b3] Rubarth J., Dreyer A., Guse N., Einax J. W. & Ebinghaus R. Perfluorinated compounds in red-throated divers from the German Baltic Sea: new findings from their distribution in 10 different tissues. Environ Chem 8, 419–28 (2011).

[b4] Wang Z., Cousins I. T., Scheringer M. & Hungerbuhler K. Fluorinated alternatives to long-chain perfluoroalkyl carboxylic acids (PFCAs), perfluoroalkane sulfonic acids (PFSAs) and their potential precursors. Environ Int 60, 242–8 (2013). 24660230 10.1016/j.envint.2013.08.021

[b5] Young C. J. *et al.* Perfluorinated acids in Arctic snow: new evidence for atmospheric formation. Environ Sci Technol 41, 3455–61 (2007). 17547163 10.1021/es0626234

[b6] Ahrens L., Shoeib M., Vento S. D., Codling G. & Halsall C. Polyfluoroalkyl compounds in the Canadian Arctic atmosphere. Environ Chem 8, 399–406 (2011).

[b7] Wang Z., MacLeod M., Cousins I. T., Scheringer M. & Hungerbuhler K. Using COSMOtherm to predict physicochemical properties of poly- and perfluorinated alkyl substances (PFASs). Environ Chem 8, 389–98 (2011).

[b8] Cousins I. T., Kong D. & Vestergren R. Reconciling measurement and modelling studies of the sources and fate of perfluorinated carboxylates. Environ Chem 8, 339–54 (2011).

[b9] Reth M. *et al.* Water-to-air transfer of perfluorinated carboxylates and sulfonates in a sea spray simulator. Environ Chem 8, 381–8 (2011).

[b10] Wang Z. *et al.* Occurrence and gas/particle partitioning of PAHs in the atmosphere from the North Pacific to the Arctic Ocean. Atmos Environ 77, 640–6 (2013).

[b11] Martin J. W., Ellis D. A., Mabury S. A., Hurley M. D. & Wallington T. J. Atmospheric chemistry of perfluoroalkanesulfonamides: kinetic and product studies of the OH radical and Cl atom initiated oxidation of N-ethyl perfluorobutanesulfonamide. Environ Sci Technol 40, 864–72 (2006). 16509330 10.1021/es051362f

[b12] Hurley M. D. *et al.* Atmospheric Chemistry of Fluorinated Alcohols: Reaction with Cl Atoms and OH Radicals and Atmospheric Lifetimes. J Phys Chem A 108, 1973–9 (2004).

[b13] Ellis D. A. *et al.* Degradation of fluorotelomer alcohols: a likely atmospheric source of perfluorinated carboxylic acids. Environ Sci Technol 38, 3316–21 (2004). 15260330 10.1021/es049860w

[b14] D'Eon J. C., Hurley M. D., Wallington T. J. & Mabury S. A. Atmospheric chemistry of N-methyl perfluorobutane sulfonamidoethanol, C4F9SO2N(CH3)CH2CH2OH: kinetics and mechanism of reaction with OH. Environ Sci Technol 40, 1862–8 (2006). 16570609 10.1021/es0520767

[b15] Piekarz A. M., Primbs T., Field J. A., Barofsky D. F. & Simonich S. Semivolatile fluorinated organic compounds in Asian and western U.S. air masses. Environ Sci Technol 41, 8248–55 (2007). 18200847 10.1021/es0713678

[b16] Wania F. A global mass balance analysis of the source of perfluorocarboxylic acids in the Arctic ocean. Environ Sci Technol 41, 4529–35 (2007). 17695892 10.1021/es070124c

[b17] Stemmler I. & Lammel G. Pathways of PFOA to the Arctic: variabilities and contributions of oceanic currents and atmospheric transport and chemistry sources. Atmos Chem Phys 10, 9965–80 (2010).

[b18] Daly G. L. & Wania F. Simulating the influence of snow on the fate of organic compounds. Environ Sci Technol 38, 4176–86 (2004). 15352458 10.1021/es035105r

[b19] Herbert B. M., Halsall C. J., Villa S., Jones K. C. & Kallenborn R. Rapid changes in PCB and OC pesticide concentrations in arctic snow. Environ Sci Technol 39, 2998–3005 (2005). 15926544 10.1021/es040076l

[b20] Shoeib M., Harner T. & Vlahos P. Perfluorinated chemicals in the arctic atmosphere. Environ Sci Technol 40, 7577–83 (2006). 17256497 10.1021/es0618999

[b21] Ahrens L., Harner T., Shoeib M., Lane D. A. & Murphy J. G. Improved characterization of gas-particle partitioning for per- and polyfluoroalkyl substances in the atmosphere using annular diffusion denuder samplers. Environ Sci Technol 46, 7199–206 (2012). 22606993 10.1021/es300898s

[b22] Dreyer A. & Ebinghaus R. Polyfluorinated compounds in ambient air from ship- and land-based measurements in northern Germany. Atmos Environ 43, 1527–35 (2009).

[b23] Xie Z. *et al.* Neutral poly- and perfluoroalkyl substances in air and seawater of the North Sea. Environ Sci Pollut Res Int 20, 7988–8000 (2013). 23636599 10.1007/s11356-013-1757-z

[b24] Wang Z. *et al.* Atmospheric concentrations and gas/particle partitioning of neutral poly- and perfluoroalkyl substances in northern German coast. Atmos Environ 95, 207–13 (2014).

[b25] Hansen K. M., Halsall C. J. & Christensen J. H. A dynamic model to study the exchange of gas-phase persistent organic pollutants between air and a seasonal snowpack. Environ Sci Technol 40, 2644–52 (2006). 16683604 10.1021/es051685b

[b26] Legagneux L., Cabanes A. & Dominé F. Measurement of the specific surface area of 176 snow samples using methane adsorption at 77 K. J Geophys Res: Atmos 107, ACH 5-1–ACH 5-15 (2002).

[b27] Lyakurwa F. S., Yang X., Li X., Qiao X. & Chen J. Development of In Silico Models for Predicting LSER Molecular Parameters and for Acute Toxicity Prediction to Fathead Minnow (*Pimephales Promelas*). Chemosphere 108, 17–25 (2014). 24875907 10.1016/j.chemosphere.2014.02.076

