# Supplementary material for: Neutral Poly-/perfluoroalkyl Substances in Air and Snow from the Arctic
Source: Sci Rep. 2015 Mar 9;5:8912. doi: 10.1038/srep08912 (PMC4352854; doi:10.1038/srep08912)
Supplement: Supplementary Information [file srep08912-s1.pdf]

Neutral Poly-/perfluoroalkyl Substances in Air and Snow from the Arctic

Zhiyong Xie[1]*, *Zhen Wang*[1,2], *Wenying Mi*[1], *Axel Möller*[1], *Hendrik Wolschke*[1], *Ralf Ebinghaus*[1]

[1]Helmholtz-Zentrum Geesthacht, Centre for Materials and Coastal Research, Institute of Coastal Research, Department for Environmental Chemistry, Geesthacht, 21502, Germany

[2]National Marine Environmental Monitoring Center, Dalian 116023, China

*Corresponding author. Phone: +49-4152-872330; Fax: +49-4152-872332;

e-mail: zhiyong.xie@hzg.de

Total: 19 pages, 6 tables and 5 figures

S1. Instrumental analysis parameters for the 12 PFASs

S2. Calculation of air-seawater gas exchange fluxes of the 12 PFASs

Table S1. Full names, acronyms, Chemical Abstract System (CAS) numbers, molecular weight (MW), structure, method detection limits (MDL) and compound descriptors of the 12 PFASs

Table S2. Detailed information on the sampling dates, the average ambient temperature (ºC), volume (m^3), total suspended particulate (TSP) ($\mu g/m^3$) for all the 45 air samples collected from Ny-Ålesund, Norwegian Arctic, and the slopes, intercepts, relationship coefficients (r^2) and significant levels (p) of the linear regression $\log K_{SP}$ vs. $\log p^{\circ}_{L}$ for each sample

Table S3. Summarized concentrations of the 12 PFASs in air and snow samples collected from Ny-Ålesund, Norwegian Arctic, and $\log p^{\circ}_{L}$ (Pa) at 25ºC

Table S4. Sampling date, the ambient temperature, concentrations (pg L^{-1}) of the 12 PFASs in snow samples from the Ny-Ålesund, the Arctic

Table S5. Air-snow exchange fluxes of the 12 PFASs (pg m^{-2} d^{-1})

Table S6. Snow interface-air partition coefficient ($\log K_{SA}$) and correlation coefficients of the fluxes with concentrations in air and snow

Figure S1. Significant correlation between $\log K_{SP}$ and $\log p^{\circ}_{L}$ (excluding FTA 6:2 and 8:2)

Figure S2. Cumulative frequency of Monte Carlo analysis (10 000 times) for the 12 PFAS compounds

Figure S3. Significant correlation between the ambient temperature and the atmospheric concentrations of FOSEs and FOSAs

Figure S4. High-volume air sampling on the platform for atmospheric observatory of German station located in Ny-Ålesund (78º55´N, 11º56´E)

Figure S5. MX Soxhlet extractor for extracting PUF/PAD-1 column and PAD-3 column

S1. Instrumental analysis parameters for the 12 PFASs

PFASs were analyzed using an Agilent 6890 GC - 5973 MS equipped with a 60 m SUPELCO WAX® 10 column (60 m × 0.25 mm × 1.0 μm) in selective ion monitoring (SIM) mode with positive chemical ionization (PCI). Methane was used as reagent gas for PCI and helium was used as carrier gas at a flow rate of 1.3 mL min^{-1}. The injection volume was 2.0 μL in the pulse splitless mode. The temperature program was as follows: initial temperature 50ºC hold for 2 min, increased to 70ºC at a rate of 3ºC/min, then increased at a rate of 10ºC/min to 130ºC, 20ºC/min to 220ºC and 120ºC/min to 275ºC, then held for 5 min. Finally, the temperature changed to 270ºC at the rate of 10ºC/min and held for 10 min.

S2. Calculation of air-seawater gas exchange fluxes of the 12 PFASs

The calculation of air-snow exchange flux for PFASs was based on the modified Whitman two-film resistance model:

$$F_{\text{gas}} = v(C_{\text{s}}/K_{\text{snow-air}} - C_{\text{a}})^{1} \quad (1)$$

where F_{gas} is the air-snow exchange flux (pg/(s m^2), C_{s} (pg/m^3) and C_{a} (pg/m^3) are the concentrations of target compounds in snow and air, respectively. $K_{\text{snow-air}}$ is the snow-air partition coefficient, and v is an exchange velocity (m/s).

$K_{\text{snow-air}}$ can be expressed by the following equation[1]:

$$K_{\text{snow-air}} = K_{\text{SA}} \text{ (SSA)} \times \rho \quad (2)$$

where K_{SA} is the snow interface-air partition coefficient, SSA is the specific surface area (m^2/kg), and ρ (kg/m^3) is the density of water[1].

The calculation of K_{SA} at -6.8ºC was based on Lei and Wania[2]:

$$\log K_{\text{SA}} \text{ (-6.8ºC)} = 3.53\Sigma\alpha_2^{\text{H}} + 3.38\Sigma\beta_2^{\text{H}} + 0.639\log L^{16} - 6.85 \quad (3)$$

where $\Sigma\alpha_2^{\text{H}}$ is the overall or summation solute hydrogen bond acidity, $\Sigma\beta_2^{\text{H}}$ is the overall or summation solute hydrogen bond basicity, and $\log L^{16}$ is the solute gas-hexadecane partition coefficient. Values of $\Sigma\alpha_2^{\text{H}}$ and $\Sigma\beta_2^{\text{H}}$ of PFASs were calculated according to Lyakurwa et al.[3], and $\log L^{16}$ were calculated using Software SPARC.

Values of K_{SA} at different ambient temperatures can be derived from the following equation: [4]

$$K_i \text{ (T}_2\text{)} = K_i \text{ (T}_1\text{)} \exp((-\Delta H_{\text{IA}}/8.314)(1/\text{T}_2 - 1/\text{T}_1)) \quad (4)$$

and ΔH_{IA} can be calculated as[2]:

$$\Delta H_{IA} = -5.07\ln K_{IA}(15^{o}C) - 108 \quad (5)$$

$$\log K_{IA}\ (15^{o}C) = 3.60\Sigma\alpha_2^{H} + 5.11\Sigma\beta_2^{H} + 0.635\log L^{16} - 8.47 \quad (6)$$

The exchange velocity v was expressed according to[1]

$$v = 1/(1/v_{air} + 1/v_{snow}) \quad (7)$$

The air side exchange velocity, v_{air}, was calculated as[1]

$$v_{air} = k^2U/(\ln(z_w/z_0)\ \ln(z_{ref}/z_0)) \quad (8)$$

where k is the von Karman's constant (0.4), U is the wind speed (m/s), z_w is the height of the wind (10 m), z_{ref} is the reference height of the wind (2 m), and z_0 is the surface roughness for snow (0.001 m).[1, 5]

The snow side exchange velocity, v_{snow}, was calculated as[1]

$$v_{snow} = D_{snow}/(d/2) \quad (9)$$

$$D_{snow} = D_{ia}\ (1-\rho_{snow}/\rho_{ice})^{1.5} \quad (10)$$

where d is snow thickness(m), and is density of ice (kg/m^3). The values of D_{ia} (cm^2/s) can be calculated based on their molar mass (M_i) as the following equation introduced by Schwarzenbach et al.[6]

$$D_{ia} = 1.55/M_i^{0.65} \quad (11)$$

Table S1. Full names, acronyms, Chemical Abstract System (CAS) numbers, molecular weight (MW), structure, method detection limits (MDL) and compound descriptors of the 12 PFASs

Full name	CAS number	Abbr.	MDL in air (pg/m^3)	MDL in snow (pg/L)	$\Sigma\alpha_2^H$ [1]	$\Sigma\beta_2^H$ [1]	$\log L^{16}$ [2] (25°C)	MW (g/mol)	Structure
6:2 Fluorotelomer alcohol	647-42-7	6:2 FTOH	0.1	1.8	0.59	0.36	2.86	364.1	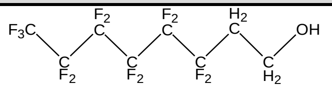
8:2 Fluorotelomer alcohol	678-39-7	8:2 FTOH	0.2	5.9	0.60	0.40	2.91	464.1	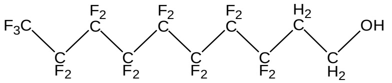
10:2 Fluorotelomer alcohol	865-86-1	10:2 FTOH	0.1	4.8	0.60	0.47	2.85	564.1	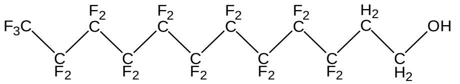
12:2 Fluorotelomer alcohol	39239-77-5	12:2 FTOH	0.1	11	0.61	0.55	2.68	664.2	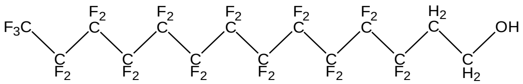
6:2 Fluorotelomer acrylate	17527-29-6	6:2 FTA	0.2	0.8	0.02	0.49	3.40	418.2	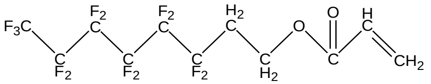
8:2 Fluorotelomer acrylate	27905-45-9	8:2 FTA	0.1	1.3	0.02	0.56	3.12	518.2	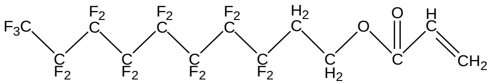
N-methyl perfluorooctane sulfonamide	31506-32-8	MeFOSA	0.1	2.3	0.33	0.90	2.94	513.2	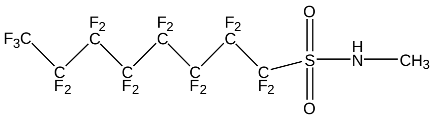
N-ethyl perfluorooctane sulfonamide	4151-50-2	EtFOSA	0.1	0.8	0.33	0.94	3.42	527.2	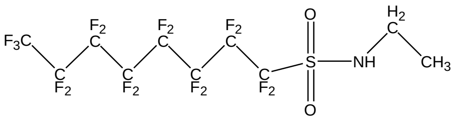

N-methyl perfluorobutane sulfonamide	68298-12-4	MeFBSA	0.1	0.1	0.31	0.84	2.69	313.1	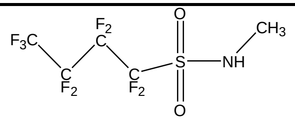
N-methyl perfluorooctane sulfonamidoethanol	24448-09-7	MeFOSE	0.2	1.5	0.61	1.21	3.82	557.2	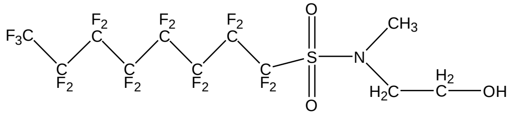
N-ethyl perfluorooctane sulfonamidoethanol	1691-99-2	EtFOSE	0.1	2.1	0.59	1.25	4.12	571.3	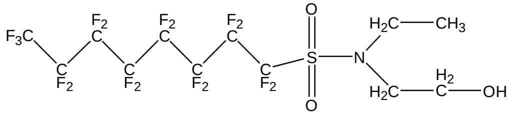
N-methyl perfluorobutane sulfonamidoethanol	34454-97-2	MeFBSE	0.1	0.2	0.58	1.12	3.57	357.2	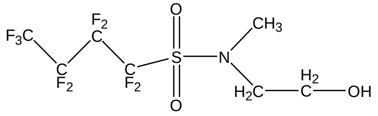

1) $\Sigma\alpha_2^H$: Over solute hydrogen bond acidity, $\Sigma\beta_2^H$: Over solute hydrogen bond basicity, calculated based on Lyakurwa et al.[3]

2) $\log L^{16}$: The solute gas-hexadecane partition coefficient, calculated using Software SPARC.

Table S2. Detailed information on the sampling dates, the average ambient temperature (ºC), volume (m^3), total suspended particulate (TSP) ($\mu g/m^3$) for all the 45 air samples collected from Ny-Ålesund, Norwegian Arctic, and the slopes, intercepts, relationship coefficients (r^2) and significant levels (p) of the linear regression $\log K_{SP}$ vs. $\log p^{\circ}_{L}$ for each sample

Samples	Sampling date	Temp. (ºC)	Volume (m^3)	TSP ($\mu g/m^3$)	Slope	Intercept	r^2	p
A1	27.9.2011	0.6	2580	5.9	-0.32	-2.25	0.74	< 0.05
A2	4.10.2011	-5.0	2539	8.5	-0.28	-1.7	0.49	< 0.05
A3	11.10.2011	-3.6	2573	20.2	-0.35	-2.28	0.84	< 0.05
A4	25.10.2011	-3.9	2505	9.6	-0.48	-1.96	0.81	< 0.05
A5	1.11.2011	-3.9	2694	2.3	-0.21	-1.81	0.83	< 0.05
A6	9.11.2011	-9.4	1613	5.6	-0.34	-2.15	0.87	< 0.05
A7	***16.11.2011***	***-6.7***	**2468**	**2.9**	***-0.18***	***-1.7***	***0.33***	***0.08***
A8	23.11.2011	-8.2	4668	20.1	-0.53	-2.33	0.85	< 0.05
A9	6.12.2011	-6.4	2529	6.4	-0.44	-1.4	0.74	< 0.05
A10	13.12.2011	-4.6	3178	3.9	-0.42	-1.64	0.74	< 0.05
A11	22.12.2011	-7.2	2888	8.6	-0.41	-1.71	0.75	< 0.05
A12	30.12.2011	-4.5	2368	10.2	-0.63	-1.67	0.86	< 0.05
A13	***5.1.2012***	***-5.8***	**2635**	**5.0**	***-0.26***	***-1.31***	***0.33***	***0.08***
A14	12.1.2012	-2.5	3193	3.6	-0.18	-1.46	0.47	< 0.05
A15	21.1.2012	-4.2	2725	2.1	-0.23	-1.52	0.51	< 0.05
A16	28.1.2012	-3.0	2139	3.6	-0.24	-1.42	0.44	< 0.05
A17	***4.2.2012***	***-7.0***	**2968**	**2.1**	***-0.18***	***-1.75***	***0.34***	***0.08***
A18	13.2.2012	-8.5	3014	3.3	-0.35	-1.66	0.68	< 0.05
A19	21.2.2012	-7.0	3016	6.3	-0.39	-1.89	0.77	< 0.05
A20	29.2.2012	-2.7	3144	7.0	-0.26	-2.2	0.74	< 0.05
A21	9.3.2012	-6.1	3584	6.2	-0.24	-1.99	0.54	< 0.05

A22	19.3.2012	-7.5	5165	7.9	-0.5	-1.78	0.8	< 0.05
A23	2.4.2012	-11.3	3434	5.2	-0.47	-1.8	0.84	< 0.05
A24	11.4.2012	-9.9	2677	4.2	-0.37	-1.69	0.81	< 0.05
A25	18.4.2012	-8.1	3031	5.9	-0.23	-1.94	0.74	< 0.05
A26	26.4.2012	-5.3	3290	12.4	-0.32	-2.31	0.83	< 0.05
A27	5.5.2012	-2.5	3309	4.7	-0.45	-1.78	0.79	< 0.05
A28	***14.5.2012***	***-1.3***	**2710**	**3.7**	***-0.15***	***-1.76***	***0.35***	***0.07***
A29	23.5.2012	1.6	2338	17.6	-0.61	-1.92	0.96	< 0.05
A30	30.5.2012	1.5	3013	10.1	-0.57	-1.94	0.82	< 0.05
A31	7.6.2012	3.1	2536	4.8	-0.48	-1.57	0.74	< 0.05
A32	13.6.2012	4.8	2652	7.2	-0.48	-2.02	0.77	< 0.05
A33	21.6.2012	4.5	2548	26.6	-0.47	-2.55	0.76	< 0.05
A34	28.6.2012	5.5	2046	22.0	-0.4	-2.45	0.67	< 0.05
A35	4.7.2012	6.1	2383	15.5	-0.36	-2.04	0.58	< 0.05
A36	11.7.2012	6.6	2506	13.1	-0.35	-2.16	0.62	< 0.05
A37	18.7.2012	6.7	2474	20.0	-0.45	-2.57	0.72	< 0.05
A38	25.7.2012	5.1	2187	3.1	-0.36	-1.32	0.49	< 0.05
A39	1.8.2012	5.0	2372	40.0	-0.47	-2.33	0.64	< 0.05
A40	8.8.2012	6.0	2594	17.3	-0.42	-2.31	0.65	< 0.05
A41	15.8.2012	4.0	2099	30.3	-0.4	-2.16	0.53	< 0.05
A42	22.8.2012	4.3	2320	9.2	-0.36	-1.64	0.49	< 0.05
A43	29.8.2012	3.3	2616	16.6	-0.4	-1.89	0.59	< 0.05
A44	14.9.2012	5.5	2324	7.4	-0.45	-1.46	0.62	< 0.05
A45	***21.9.2012***	***-1.7***	**2077**	**21.0**	***-0.18***	***-2.17***	***0.3***	***0.1***

Table S3. Summarized concentrations of the 12 PFASs in air and snow samples collected from Ny-Ålesund, Norwegian Arctic, and logp°_{L} (Pa) at 25ºC

Compounds	logp°_{L} (Pa, 25ºC)	air (pg m^{-3})					snow (pg L^{-1})				
		ave.	std	max	min	%ΣPFASs	ave.	std	max	min	%ΣPFASs
**6:2 FTOH**	1.94	2.1	0.8	5.1	0.6	12.7	4.2	1.6	6.6	1.5	0.8
**8:2 FTOH**	1.61	10	4.0	25	3.7	60.8	236	82	360	114	45.1
**10:2 FTOH**	1.35	2.0	1.6	9.3	0.4	12.0	95	53	148	20	18.2
**12:2 FTOH**	1.09	0.8	0.6	3.3	0.3	4.8	34	16	72	15	6.5
**6:2 FTA**	1.67	0.1	0	0.1	< 0.1	0.0	3.9	1.8	6.4	1.4	0.7
**8:2 FTA**	1.34	0.1	0.1	0.4	< 0.1	0.6	5.3	2.5	8.7	1.7	1.0
**MeFOSA**	1.18	0.3	0.1	0.5	0.1	1.8	5.3	4.4	17	2.3	1.0
**EtFOSA**	0.82	0.2	0.1	0.5	0.1	1.2	4.0	2.0	7.8	1.4	0.8
**MeFBSA**	1.95	0.6	0.5	1.8	0.1	3.6	26	22	57	0.8	5.0
**MeFOSE**	-1.39	0.2	0.2	0.8	< 0.1	1.2	57	29	96	17	10.9
**EtFOSE**	-1.76	0.1	0.1	0.4	< 0.1	0.6	23	13	43	7.1	4.4
**MeFBSE**	-0.62	0.1	0	0.2	< 0.1	0.6	29	7.4	41	19	5.5
**ΣPFASs**		17	6.3	39	6.7		523	141	692	334	

Table S4. Sampling date, the ambient temperature, concentrations (pg L^{-1}) of the 12 PFASs in snow samples from the Ny-Ålesund, the Arctic

samples	FTOH 6:2	FTOH 8:2	FTOH 10:2	FTOH 12:2	FTA 6:2	FTA 8:2	MeFOSA	EtFOSA	MeFBSA	MeFOSE	EtFOSE	MeFBSE	sum	date	*t* (°C)
S1	4.8	146	139	27	6.4	8.4	3.6	1.4	5.4	56	23	32	454	2012/1/31	-1.6
S2	1.5	114	87	15	1.4	1.7	4.4	3.5	10	69	28	24	361	2012/1/31	-1.6
S3	4.8	251	148	25	4.9	3.7	3.3	2.1	52	76	26	27	624	2012/2/2	-3.4
S4	6.6	237	148	31	5.7	3.5	3.7	4.8	57	94	43	30	662	2012/2/4	-3.1
S5	4.8	314	135	23	2.5	8.1	3.5	3.7	23	96	41	37	692	2012/5/18	-1.5
S6	2.6	211	22	32	2.4	3.8	2.3	2.4	7.7	17	11	19	334	2012/5/21	-1.5
S7	3.5	310	20	42	3.2	5.1	5.2	5.6	41	39	18	41	533	2012/5/23	-1.6
S8	5.9	183	45	72	5.4	8.7	4.6	5.0	0.8	30	12	20	392	2012/5/24	-0.5
S9	3.2	360	105	39	2.9	4.7	17	7.8	41	35	7.1	30	652	2012/5/22	-1

Table S5. Air-snow exchange fluxes of the 12 PFASs (pg m^{-2} d^{-1})

samples	6:2 FTOH	8:2 FTOH	10:2 FTOH	12:2 FTOH	6:2 FTA	8:2 FTA	MeFOSA	EtFOSA	MeFBSA	MeFOSE	EtFOSE	MeFBSE	Sum
S1	1.22	49.72	28.96	3.19	66.05	68.03	0.15	-0.02	0.99	-0.03	-0.01	-0.05	218
S2	-1.09	37.70	17.93	1.64	14.11	14.16	0.20	0.03	2.13	-0.02	-0.01	-0.05	87
S3	1.17	88.55	30.83	2.92	50.67	30.03	0.12	-0.01	11.77	-0.02	-0.01	-0.05	216
S4	2.42	83.31	30.70	3.60	59.23	28.61	0.15	0.06	12.97	-0.02	-0.01	-0.05	221
S5	1.98	96.01	24.38	2.31	25.57	65.61	-0.35	-0.24	4.74	-0.07	-0.03	-0.19	220
S6	0.44	57.37	0.66	3.43	24.75	30.85	-0.42	-0.27	1.14	-0.08	-0.03	-0.20	118
S7	-1.94	99.55	-0.04	4.52	33.00	41.37	0.08	-0.02	7.65	-0.05	-0.02	-0.04	184
S8	2.72	46.89	5.53	8.56	56.30	70.40	-0.28	-0.22	-0.46	-0.08	-0.03	-0.20	189
S9	-2.12	118.06	17.86	4.09	30.41	38.12	0.85	0.03	7.60	-0.05	-0.02	-0.05	215

Table S6. Snow interface-air partition coefficient (logK_{SA}) and correlation coefficients of the fluxes with concentrations in air and snow

Compounds	logK_{SA}	r^2_a*	r^2_s*
6:2 FTOH	-1.71	0.32	0.63
8:2 FTOH	-1.52	0.10	0.95
10:2 FTOH	-1.32	0.54	0.99
12:2 FTOH	-1.15	0.41	0.99
6:2 FTA	-2.93	0.24	0.99
8:2 FTA	-2.88	0.01	0.99
MeFOSA	-0.79	0.37	0.71
EtFOSA	-0.33	0.86	0.13
MeFBSA	-1.20	0.03	0.98
MeFOSE	1.82	0.96	0.19
EtFOSE	2.10	0.98	0.10
MeFBSE	1.29	0.99	0.02

*r^2_a: correlation coefficients of the fluxes with concentrations in air.

r^2_s: correlation coefficients of the fluxes with concentrations in snow.

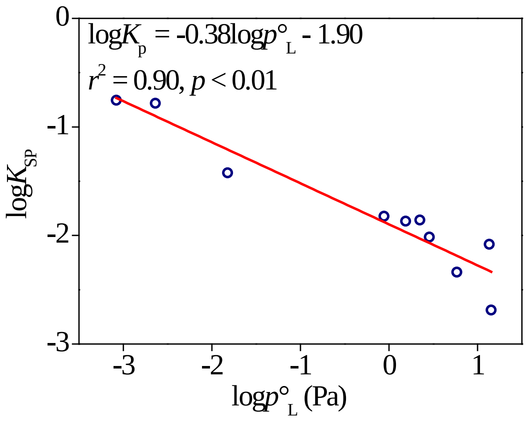

Figure S1. Significant correlation between $\log K_{SP}$ and $\log p^{\circ}_{L}$ (excluding FTA 6:2 and 8:2)

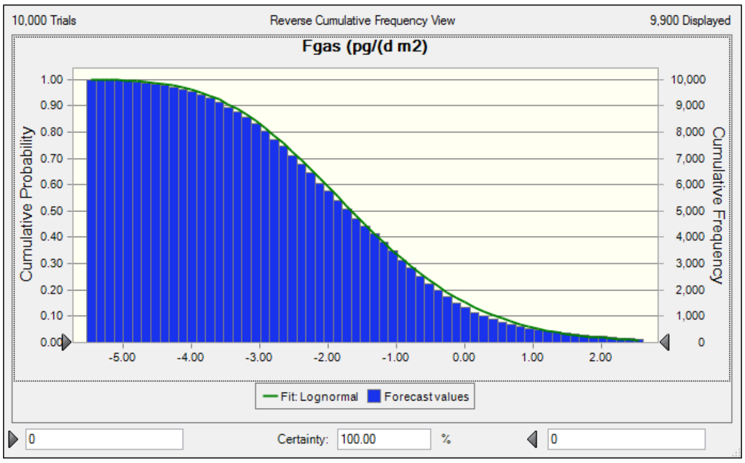

6:2 FTOH

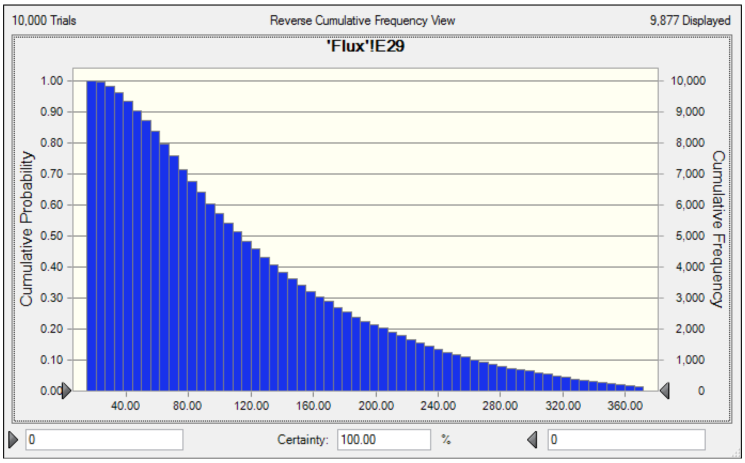

8:2 FTOH

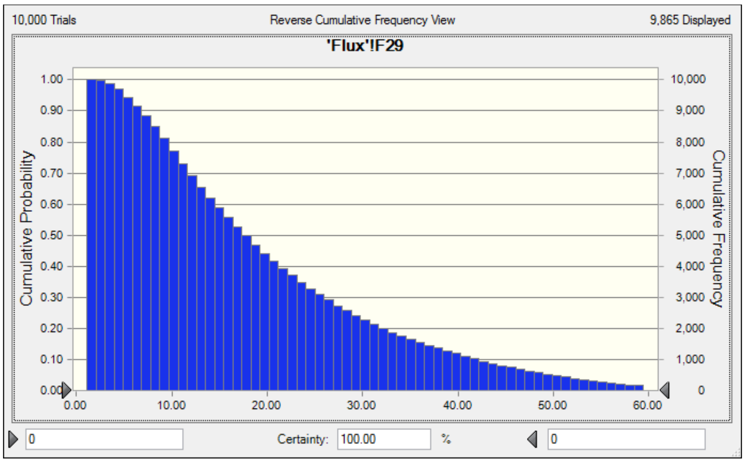

10:2 FTOH

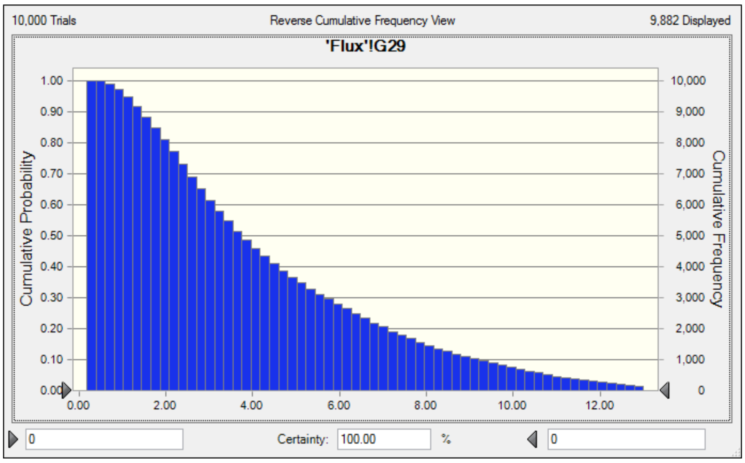

12:2 FTOH

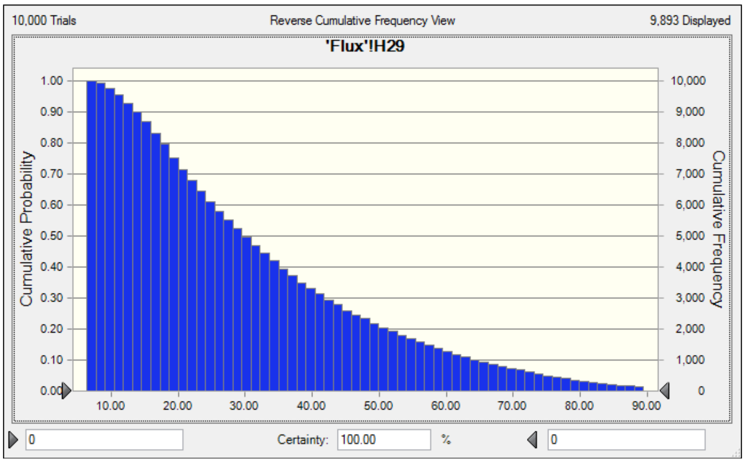

6:2 FTA

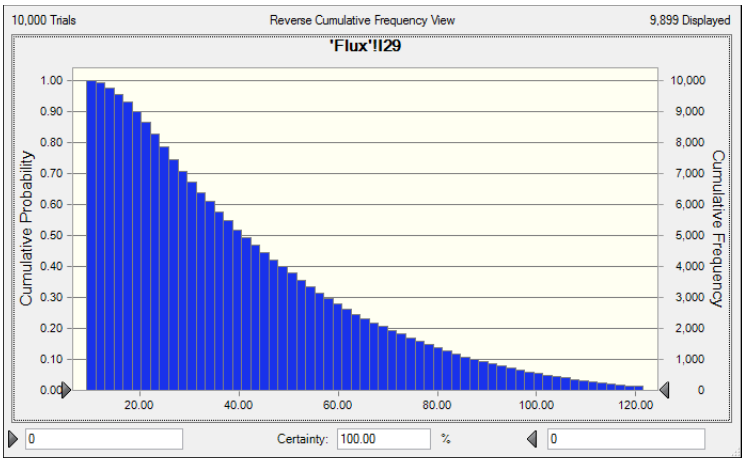

8:2 FTA

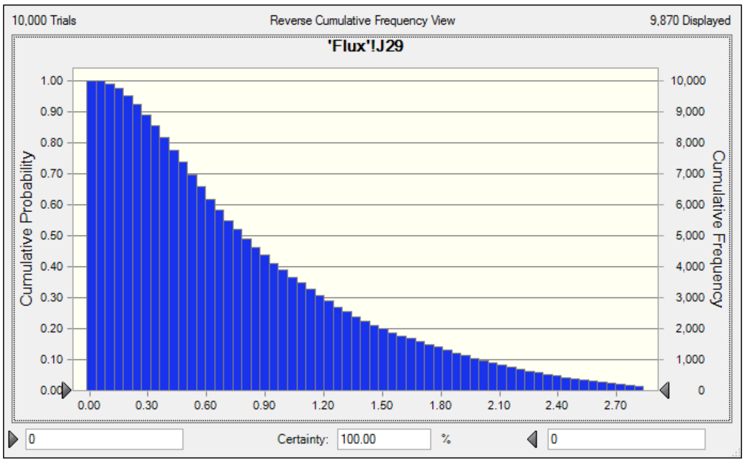

MeFOSA

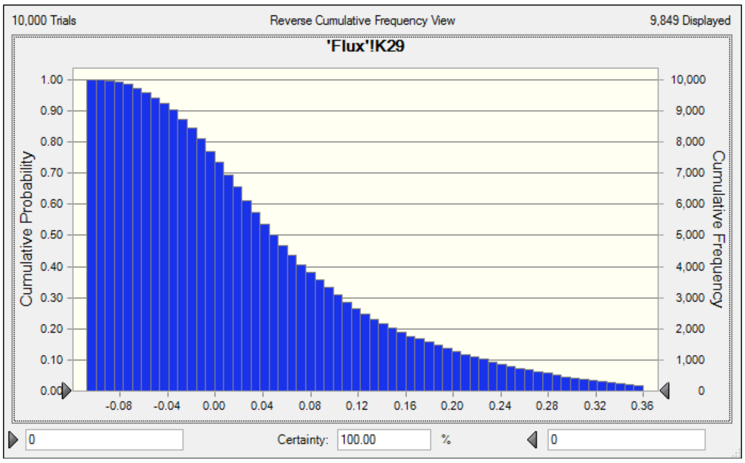

EtFOSA

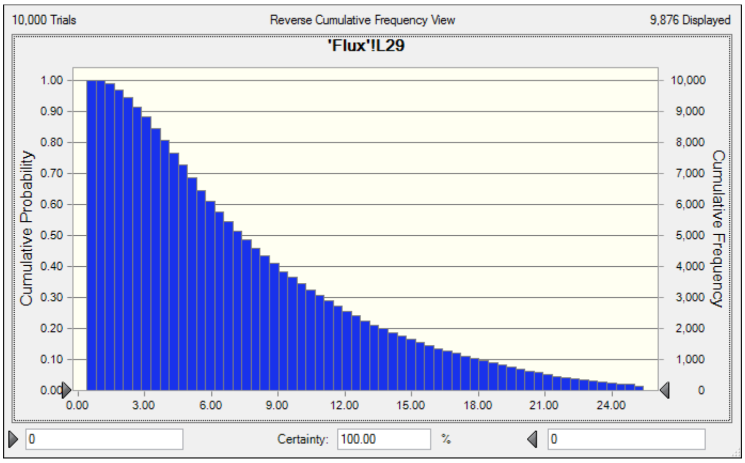

MeFBSA

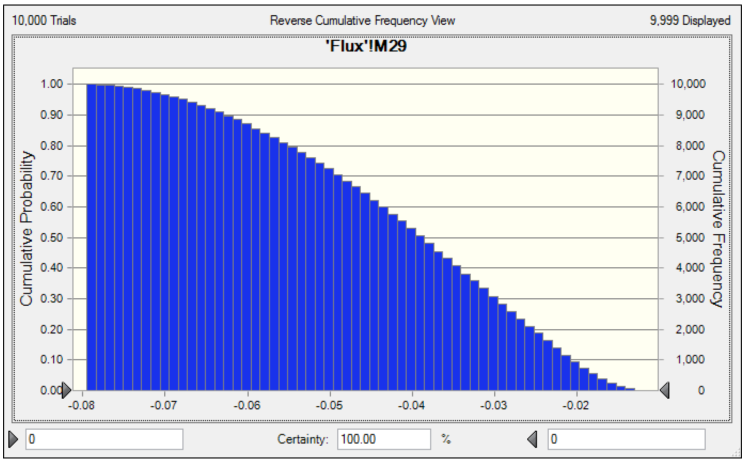

MeFOSE

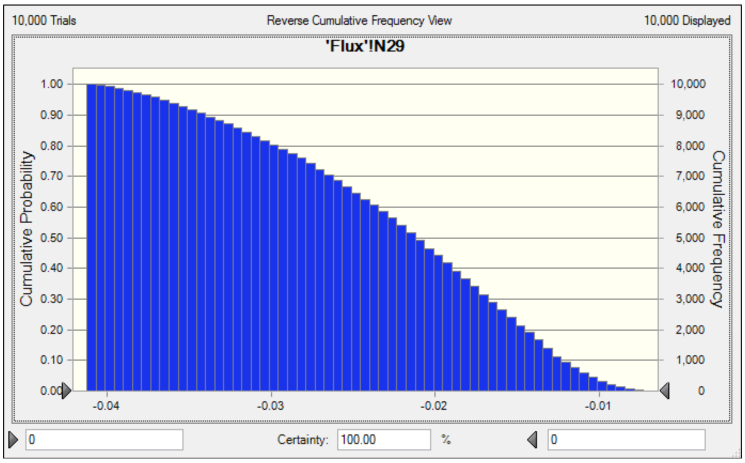

EtFOSE

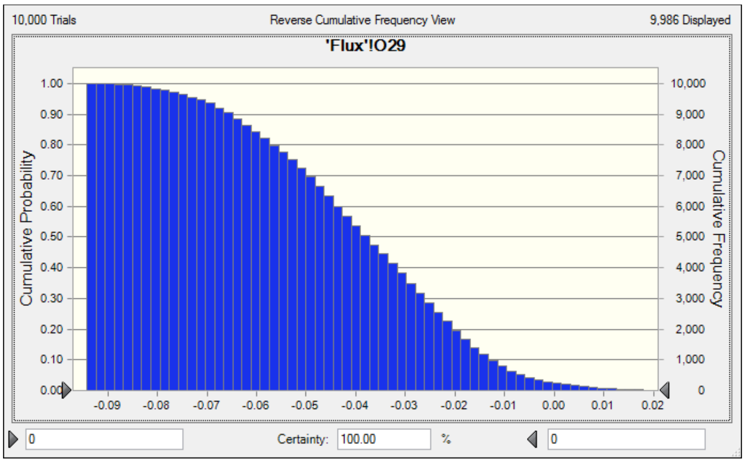

MeFBSE

Figure S2. Cumulative frequency of Monte Carlo analysis (10 000 times) for the 12 PFAS compounds

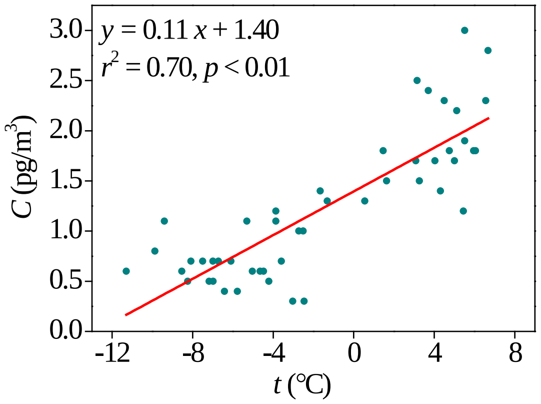

Figure S3. Significant correlation between the ambient temperature and the atmospheric concentrations of FOSEs and FOSAs

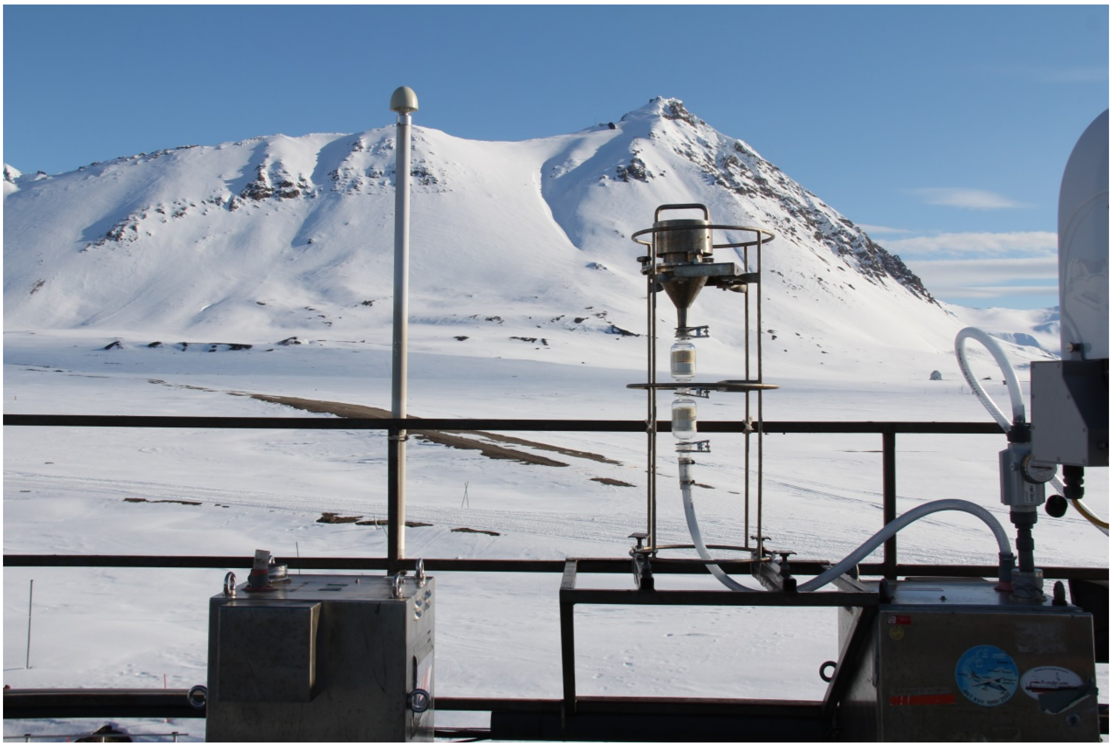

Figure S4. High-volume air sampling on the platform for atmospheric observatory of German station located in Ny-Ålesund (78°55´N, 11°56´E)

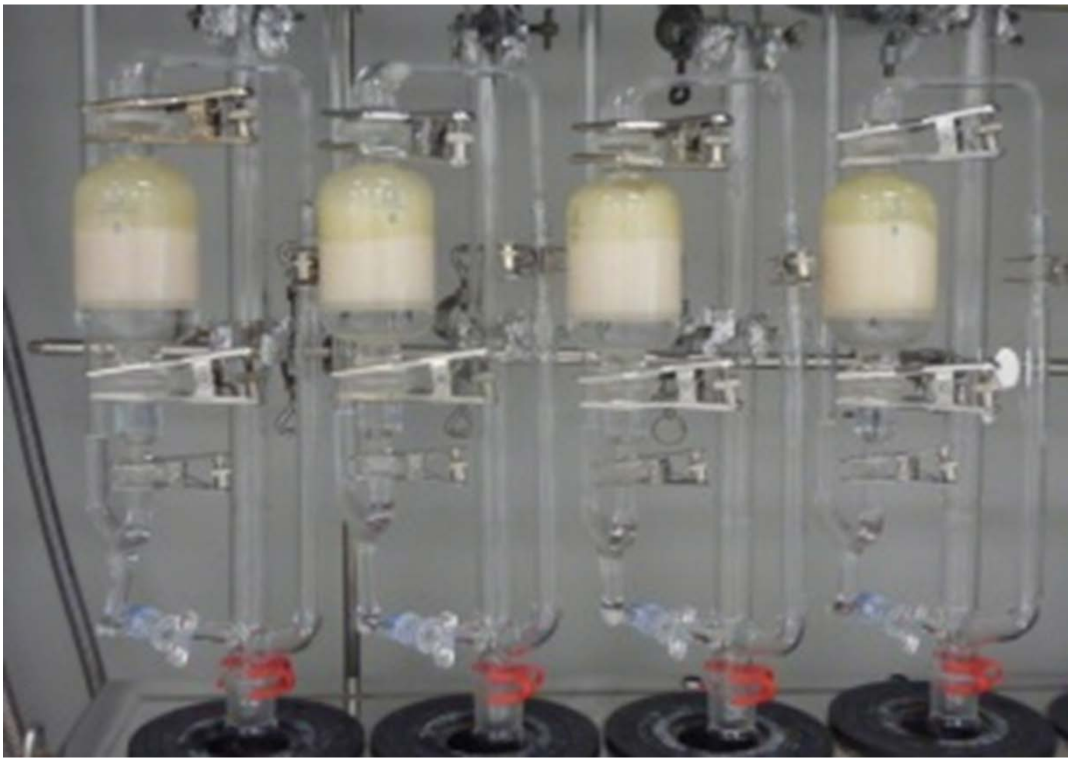

Figure S5. MX Soxhlet extractor for extracting PUF/PAD-1 column and PAD-3 column.

References

1. Hansen KM, Halsall CJ, Christensen JH. A dynamic model to study the exchange of gas-phase persistent organic pollutants between air and a seasonal snowpack. *Environmental science & technology* **40**, 2644-2652 (2006).

2. Lei YD, Wania F. Is rain or snow a more efficient scavenger of organic chemicals? *Atmospheric Environment* **38**, 3557-3571 (2004).

3. Lyakurwa FS, Yang X, Li X, Qiao X, Chen J. Development of In Silico Models for Predicting LSER Molecular Parameters and for Acute Toxicity Prediction to Fathead Minnow (Pimephales Promelas). *Chemosphere* **108**, 17-25 (2014).

4. Schwarzenbach RP, Gschwend PM, Imboden DM. Environmental organic chemistry (Second Edition) [M]. New Jersey: A John Wiley & Sons, Inc. Eq. 3-51 (2003).

5. Seinfeld JH, Pandis SN. Atmospheric chemistry and physics: From air pollution to climate change; John Wiley & Sons: New York. (1998).

6. Schwarzenbach RP, Gschwend PM, Imboden DM. Environmental organic chemistry (Second Edition) [M]. New Jersey: A John Wiley & Sons, Inc. Fig. 18.19 (2003).
